# Nutrient, pigment, suspended matter and turbidity measurements in the Belgian part of the North Sea

**DOI:** 10.1038/s41597-019-0032-7

**Published:** 2019-04-09

**Authors:** Jonas Mortelmans, Klaas Deneudt, André Cattrijsse, Olivier Beauchard, Ilse Daveloose, Wim Vyverman, Jan Vanaverbeke, Klaas Timmermans, Jan Peene, Patrick Roose, Mark Knockaert, Lei Chou, Richard Sanders, Marc Stinchcombe, Philippe Kimpe, Saskia Lammens, Hannelore Theetaert, Thanos Gkritzalis, Francisco Hernandez, Jan Mees

**Affiliations:** 1 Flanders Marine Institute (VLIZ), Wandelaarkaai 7, 8400 Oostende, Belgium; 2 Ghent University, Laboratory of Protistology & Aquatic Ecology (PAE), Krijgslaan 281, 9000 Ghent, Belgium; 3 Ghent University, Marine Biology Research Group (MARBIOL), Krijgslaan 281, 9000 Ghent, Belgium; 4 Royal Netherlands Institute for Sea Research (NIOZ), Department of Estuarine and Delta Systems, and Utrecht University, PO Box 140, 4401 NT Yerseke, The Netherlands; 5 Royal Belgian Institute of Natural Sciences, Operational Directorate Natural Environment (OD Nature), Vautierstraat 29, 1000 Brussels, Belgium; 6 Université Libre de Bruxelles, Service de Biogéochimie et Modélisation du Système Terre, CP208, Boulevard du Triomphe, 1050 Bruxelles, Belgium; 7 Ocean Biogeochemistry and Ecosystems, National Oceanography Centre (NOC), European Way, SO14 3ZH Southampton, United Kingdom; 8 Vlaamse Milieumaatschappij (VMM), Zandvoordestraat 375, 8400 Oostende, Belgium; 9 Present Address: University of Antwerp, Department of Biology, Ecosystem Management Research Group (ECOBE), Universiteitsplein 1, 2610 Wilrijk, Belgium; 10 Present Address: Royal Belgian Institute of Natural Sciences, Operational Directorate Natural Environment (OD Nature), Marine Ecology and Management (MARECO), Vautierstraat 29, 1000 Brussels, Belgium

**Keywords:** Databases, Marine biology, Water resources, Marine chemistry

## Abstract

Through regular sampling surveys, the Flanders Marine Institute is generating long term data series for the Belgian coastal water and sand bank systems, a designated site in the Long Term Ecological Research (LTER) network. The data series is built on sampling activities initiated in 2002, but gradually upgraded and extended in the framework of the LifeWatch marine observatory and the Integrated Carbon Observation System (ICOS) participation. Nine nearshore stations are sampled monthly, with additional seasonal sampling of eight offshore stations. This paper presents the generated data series for nutrients, pigments, suspended matter and turbidity. The collection, methodology and processing of the 2002–2018 dataset is described, along with its data curation, integration and quality control. Yearly versions of the data are published online in a standardized format, accompanied with extensive metadata description and labelled with digital identifiers for traceability. Data is published under a CC-BY license, allowing use of the data under the condition of providing reference to the original source.

## Abstract (summary)

**Table:** 

Design Type(s)	source-based data analysis objective • data collection and processing objective • observational design
Measurement Type(s)	pigment • nutrient • waterborne particulate matter • Turbidity Measurement
Technology Type(s)	high pressure liquid chromatography • segmented flow analyzer • balance • Secchi disk
Factor Type(s)	temporal_interval
Sample Characteristic(s)	North Sea • sea

Machine-accessible metadata file describing the reported data (ISA-Tab format)

## Background & Summary

Large environmental changes, either natural or anthropogenic, generally occur over long periods of time. Physico-chemical properties of water are known to follow periodic seasonal changes, but ongoing climate changes affecting temperature, primary production and species distributions may drive ecosystem shifts over the long term^1^. Therefore, it is crucial to monitor the environmental conditions in coastal waters consistently over a long period of time. For this purpose, the Flanders Marine Institute (VLIZ) initiated a scheme of monthly sampling campaigns in the Belgian Part of the North Sea (BPNS) in 2002.

The BPNS is part of the Southern Bight of the North Sea and covers 3,447 square kilometres. The area is characterized by a shallow depth (maximum 40 m) and a series of subtidal sand bank systems^2^. Water masses in the BPNS are strongly influenced both by saline waters from the English Channel and freshwater inputs from river discharges (e.g. Ijzer, Scheldt, Maas)^3,4^. Especially the Scheldt estuary exerts a dominant influence, regarding the suspended matter, fauna and flora^4,5^. The Belgian coastal waters and sand bank systems are a designated site in the LTER Network (https://lternet.edu/), a network aiming to identify drivers of global, regional and local ecosystem changes through the collection of long-term data series.

Consistent long term data series for the Belgian coastal waters were lacking before the 1980’s. Available *in-situ* data for nutrients, pigments, suspended matter and turbidity were gathered during targeted expeditions and projects^2^ and as such, are limited in spatial and temporal coverage. In the framework of the 4DEMON project, an extensive overview is created to concatenate these historical datasets^6,7^. From 1976 onwards, the Management Unit of the North Sea Mathematical Models (MUMM) initiated a more continuous monitoring effort for environmental variables. This monitoring focuses on the quality evaluation of the marine environment to fulfil policy needs and reporting obligations in the framework of Oslo and Paris convention (OSPAR)^8^, the Water Framework Directive (WFD)^9^ and the Marine Strategy Framework Directive (MSFD)^10^. From 2002 onwards, these monitoring activities are complemented with the sampling surveys organized by VLIZ, described in this paper. These sampling campaigns were initiated by VLIZ in collaboration with the Laboratory of Protistology & Aquatic Ecology (PAE, Ghent University), Marine Biology Research Group (MARBIOL, Ghent University), the Flanders Environment Agency (VMM), the Royal Netherlands Institute for Sea Research (NIOZ), the Royal Belgian Institute of Natural Sciences, OD Nature (OD Nature, RBINS) and the National Oceanography Centre Southampton (NOC). In the framework of the Flemish contribution to ESFRI research infrastructures LifeWatch and ICOS, VLIZ intensified the campaigns in 2012.

During the multi-disciplinary sampling campaigns, water column data is collected on physical, biochemical and biodiversity related aspects of the environment, including measurements of nutrients, pigments, suspended matter and turbidity. These water quality descriptors can provide supporting information for status assessments of eutrophication, pollution and changes in coastal waters^11–14^. Nutrients are an important resource at the basis of the food chain and are mainly consumed by phytoplankton and bacteria. Greatly influenced by wastewater inputs and diffuse input from agriculture, disturbed nutrient concentrations may cause problems of eutrophication^15^. This dataset holds 9,343 nutrient records collected between August 2002 and December 2018. Pigment concentrations have widely been used as taxonomic markers within the marine environment^16^. These concentrations are increasingly used in plankton research for the quantification of major taxonomic groups of phytoplankton (e.g. Wright and Jeffrey^17^ and references therein). Here, 24 types of pigment are measured, resulting in 30,668 records between August 2002 and December 2018, of which chlorophyll a, chlorophyll b, chlorophyll c3, fucoxanthin and diadinoxanthine are the most common ones. Finally, turbidity affects the penetration of light into the water column and influences primary production. Secchi depth and suspended particulate matter (SPM) are both used as a proxy for turbidity. Values for Secchi depth are *in-situ* estimates of water transparency, whereas SPM is determined through laboratory measurements of particulate matter concentration, both organic and inorganic. Available Secchi depth and SPM accounted for respectively 1,619 and 1,540 records for the period August 2002 and December 2018. An overview of all available parameters is found in Online only Table 1.

## Methods

The entire pathway from sampling towards realising online data accessibility is described below. Since 2016, upgraded versions of the database are processed annually: the incremental dataset versions including the sampling data up to 2016^18^, 2017^19^ and 2018^20^ are available through the Marine Data Archive (http://mda.vliz.be/) and the LifeWatch Data explorer (http://www.lifewatch.be/en/lifewatch-data-explorer). These datasets are described in an ISO-19115 compliant catalogue of the Integrated Marine Information System (IMIS) and labelled with Digital Object Identifiers for traceability.

### Sampling design

**Fig. 1:**
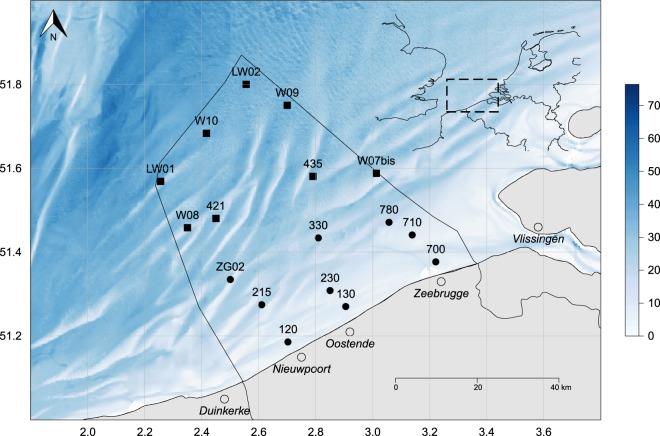
Map of the sampling area. Top-right insert indicates location of the study area in the southern North Sea. The colour bar represents the bathymetry in meters. Black circles are the nearshore sampling stations (monthly monitored); black squares are the off-shores sampling stations (seasonally monitored). The polygon delineates the Belgian exclusive economic zone.

In total, 17 stations in the BPNS are being sampled with a regular frequency: nine stations close to shore on a monthly basis and eight additional stations located further offshore on a seasonal basis (Fig. 1). The locations of these stations, more or less evenly distributed over the BPNS, were chosen in 2002 based on the availability of historical data, as well as for reasons of complementarity with the monitoring by OD Nature (RBINS). Because of logistical and budgetary restrictions, the offshore stations could only be visited on a seasonal basis, generally four times a year. Sampling at these stations was initiated in 2012.

### Sampling methods

**Fig. 2:**
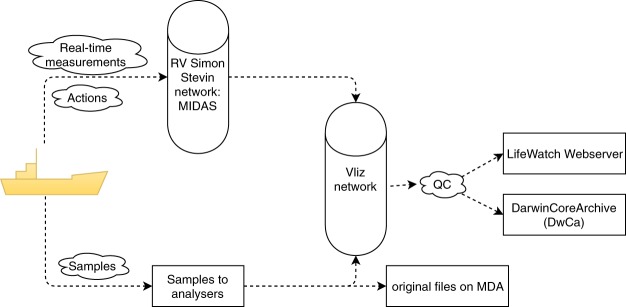
Schematic overview of the data flow from ship to user. The data flow of both actions and samples are illustrated, both data flows come together in the VLIZ network prior to dissemination to the broad public.

Prior to 2012, all measurements were carried out aboard the research vessel (RV) Zeeleeuw and since 2012, the aboard the RV Simon Stevin. When at sea, the Marine Information and Data Acquisition System (MIDAS) registers the navigation data (including heading, current time, latitude, longitude, speed and course over ground, navigation depth and draught) as well as meteorological (air temperature and relative humidity, wind direction and speed) and oceanographic data (sea surface water temperature, salinity, chlorophyll a concentrations and sound velocity). This application enables marine scientists to log their research activities during each scientific campaign. Specific actions on board are registered on the spot and the related metadata are made available online every 24 h through an automated synchronization to the VLIZ ICT network. Details on researchers, trips and cruises are stored, together with metadata from onboard research activities called ‘actions’ (e.g. time and geographical location of start and stop of scientific activities, notes, station, action type, status of deployment) (Fig. 2). The system also aids to plan cruises and register ship activities.

All water samples analysed in this dataset are collected using 5-liter Niskin bottles attached to a CTD carousel. The Niskin bottles are closed at three-meter depth and the sampled water are treated and prepared for storage in three ways:

- Pigments. Throughout the entire data series, only one protocol is used for pigment analysis. A vacuum pump and filter unit, in combination with Whatman GF/F glass fibre filters (47 mm) is used. As much seawater as possible is filtered up to saturation of the filter. The filtered amount is consistently registered in the MIDAS system for a posteriori calculation of pigment concentration. For coastal stations, this is generally limited to around 500 mL in total, whereas around 2,000 mL or more is processed at offshore stations. Once the filter runs dry, the sides of the sample container are flushed clean with Milli-Q water. The filter is folded, dried on paper tissue and stored in a 2 mL storage unit and finally labelled. The unit is stored in liquid nitrogen. Afterwards, all used equipment is rinsed thrice with Milli-Q water.
- Nutrients. Two slightly different protocols are used, depending on the laboratory performing the analysis. In both cases, around 200 mL of seawater is filtered through a 47 mm, 0.2 µm cellulose-acetate filter for residual water. When the filter runs dry, 150 mL of filtered water is poured into a recipient and then stored at −24 °C. The Erlenmeyer, recipients and all other equipment are rinsed thrice with Milli-Q water. After the cruise, all samples are transferred directly to the Marine Station Oostende (MSO) and stored at their appropriate temperature again. Pre-treatment for analysis by one of the laboratories (VMM) required the additional step of pouring 150 ml of seawater in a pre-labelled recipient rinsed with acid, to avoid potential contamination from the recipient.
- Suspended Particulate Matter. Throughout the entire data series, only one protocol on board is used for determination of Suspended Particulate Matter concentration (SPM). One litre of unfiltered seawater from the Niskin bottles, closed at 3 m depth, is taken and poured in a labelled recipient and stored at 4 °C. After the cruise, all recipients are transferred directly to the MSO and stored at their appropriate temperature again.
- Secchi disk measurements are taken from the side of the ship, practicing a method that remained identical during the whole data series: a 30 cm diameter, white Secchi disk is lowered in the water. The disk is lowered into the water until invisible, then hauled up again^21,22^. The depth at which the disk becomes visible to the researcher is noted in MIDAS.

### Quantification methods

Pigment samples are stored and processed in batches at regular intervals, generally four times a year. During the whole data series, High Pressure Liquid Chromatography (HPLC) is used for the determination of pigments although three slightly different protocols were used. Full analytical methods were described^16,23,24^ and summarized in Table 1. Nutrient samples are stored and processed in batches at regular intervals, generally four times a year. Two laboratories analysed the samples by means of a SEAL QuAAtro analysis system (NOCS, NIOZ), three laboratories by means of discrete analysis system and spectrophotometric detection with a Skalar AutoAnalyser system (VMM, ULB, OD Nature). The determination of suspended matter is done by filtration through a glass-fibre filter with a density between 50–100 g/m², and the amount of dried residue after dehydration, is measured.

**Table 1:** Summary of analysing institutes, and time periods active.

Descriptor	Laboratory	First date	Last date	Reference
Pigment	Ugent – PAE	21/8/2002	9/12/2003	^16^
		21/1/2004	8/12/2004	^23^
		17/1/2005	18/8/2008	^16^
		26/9/2008	Current	^24^
Nutrient	ULB	21/8/2002	9/12/2003	
	OD Nature	21/1/2004	23/7/2007	
	VMM	26/1/2009	26/2/2013	
	NOC	18/11/2010	29/8/2013	
	NIOZ	25/9/2013	Current	
SPM	ULB	21/8/2002	9/12/2003	
	OD Nature	21/1/2004	23/7/2007	
	VMM	26/1/2009	Current	
Turbidity	VLIZ	21/8/2002	Current	

## Data Records

The original spreadsheet files from analytical laboratories are stored in the MDA and copied to a network archive within VLIZ, where they are backed up every 24 h and linked to the corresponding research action records registered in MIDAS. These data are imported into an SQL database to allow data manipulation, quality control, visualisation and the re-distribution through an online interface. Data is disseminated in three ways:

1. On a yearly basis, data are exported from the server database and stored in an online and open-access repository formatted in compliance with the OBIS-ENV DATA standard^27^, including quality flags. This standard is considered as the most suitable format for sample based non-biological data and uses the Darwin Core Archive (DwC-A) for packaging components of Darwin Core biodiversity information in a single, self-contained dataset. The information related to sampling time and space are stored into a single text file called “Event Core”, whereas sampling descriptions and measured values are stored in another text file called “Extended MeasurementOrFactExtension” or “eMoF”. Within this format, all data are linked to domain-specific controlled vocabularies developed by the British Oceanographic Datacentre (BODC, https://www.bodc.ac.uk/resources/products/web_services/vocab/). These vocabularies are accessible web services (P01 for identifying marine environmental and biological measurements, P06 to identify units and L22 for defining sensors and instruments). Since 2016, this dataset is published on a yearly basis and is given a digital object identifier^18–20^.
2. Regular updates of the dataset are disseminated through the SeaDataNet (SDN) infrastructure (https://www.seadatanet.org/Data-Access), operating the Common Data Index (CDI) to describe metadata and Ocean Data View (ODV) to contain the data itself, and associated quality flags.
3. Via the LifeWatch data explorer, it is possible to browse quality-controlled data (labelled with ‘Under detection limit’, ‘Good data’ or ‘Probably Good data’), select on specific water quality descriptors, specify temporal and spatial windows and create exports of that data (http://www.lifewatch.be/en/lifewatch-data-explorer).
4. Via the LifeWatch data explorer, it is also possible to associate additional data on coastal tides, offshore tides, fraction of the moon’s disk that is illuminated and solar angle. These additional data enable researchers to fully exploit the effect on moon and tidal phases on the described data.

## Technical Validation

### Quality control

Since the quality of data depends on ubiquitous variables (e.g. sampling protocol, different analytical laboratories, methods, storage, shipping, etc.) potentially resulting in erroneous measurements, it is essential to perform a rigid quality control to enable a systematically comparable and correct dataset. In this dataset, eight consecutive steps are taken to assess data quality, resulting in specific quality flags associated with each measurement. The quality flags used in this dataset are found in the L20 controlled vocabulary developed by the BODC

- Good data (62.73% of all measurements)
- Values below detection limit (30.17% of all measurements)
- Probably good data (0.70% of all measurements)
- Probably bad data (4.95% of all measurements)
- Bad data (0.83% of all measurements)
- Missing values (0.62% of all measurements)

The consecutive quality control steps are described below, and are executed in this specific order:

- Values below the detection limit of the measuring instrument were flagged ‘value below detection limit’, otherwise left blank.
- Data supplied by specific providers proved resulting from inadequate quantification methods for seawater concentrations are flagged ‘Probably bad data’, otherwise left blank.
- If the geographic coordinates of the samples are found to be outside the trajectory of the RV Simon Stevin or RV Zeeleeuw at that moment, data are flagged ‘Probably bad data’, otherwise left blank.
- Global minimum and maximum values of each variable are assessed, in order to identify impossible measurements that are due to uncalibrated or broken instruments. If measurements are outside these ranges a flag ‘bad data’ is given. If within the range, the flag is left blank.
- Regional minimum and maximum values are assessed for each parameter based on datasets of Rijkswaterstaat (RWS; https://waterinfo.rws.nl/#!/nav/index/) on the Netherlands (region bordering Belgian Waters) and OD Nature (http://www.bmdc.be/NODC/index.xhtml) on the BPNS. If measurements are outside these regional ranges a flag ‘probably bad data’ is given. If within the range, the flag is left blank.
- A specific step is taken to assess whether values for a certain variable, collected during one trip can be considered as an outlier (further named ‘Trip Outlier’). Values for each parameter across all stations are compared. Values above a threshold value of 4 times the standard deviation are marked as ‘probably good data’, otherwise left blank.
- A next step is taken to assess whether values for a certain parameter, collected over a period of three months (independent of the trip), can be considered as outlier (further named ‘Temporal Outlier’). Values for each parameter, in a timeframe of three months, are compared. Values above a threshold of 4 times the standard deviation are marked as ‘probably good data’.
- The last step includes approval of the data. All remaining values without a flag are considered good data and are labelled as such.

### Spatio-temporal data availability

**Fig. 3:**
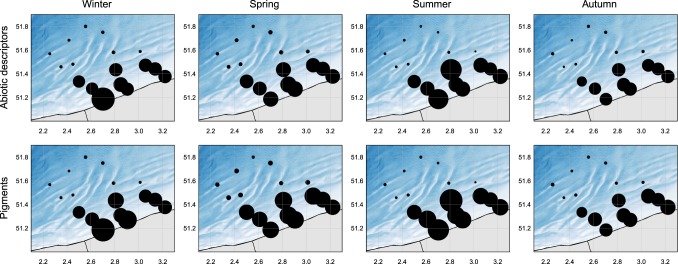
Spatio-temporal data availability in the sampled area. The size of the dots are proportional to the number of measurements at the given sampling station (smaller dots indicate fewer amounts of measurements, larger dots indicate higher amount of measurements). For abiotic descriptors, the maximum size of a dot is equal to 329 measurements per season, per station. For pigments, the maximum size a dot is equal to 897 measurements per season, per station.

**Fig. 4:**
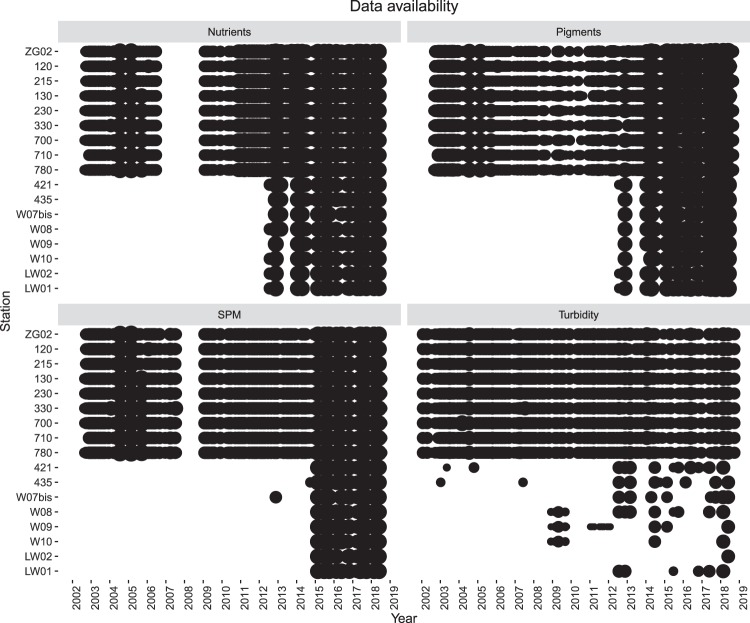
Temporal data availability in the sampled area. The figure represents all data over the 17 stations, grouped per parameter and per trip. Size of the dots are proportional to the number of measurements per trip (smaller dots indicate fewer amounts of measurements for that trip, larger dots indicate higher amount of measurements for that trip). For nutrients, the maximum size of a dot is equal to 120 measurements per trip; for pigments, the maximum size of a dot is equal to 374 measurements per trip; for SPM, the maximum size of a dot is equal to 23 measurements per trip; for turbidity, the maximum size of a dot is equal to 19 measurements per trip.

The spatio-temporal data availability is heterogeneous and depending on the spatio-temporal selection. Discrepancies in availability may occur, especially between nearshore and offshore stations. This discrepancy is mainly due to the design of the sampling campaigns, with onshore stations being sampled with a higher frequency (generally 12 times a year) than the offshore stations (generally 4 times a year) (Fig. 3). A second reason, enforcing the first, is due to the fact that offshore stations have been visited only since 2012 (nutrients and pigments), 2014 (SPM), or on an irregular basis (turbidity), generating an important shift between 2012 and 2014 (Fig. 4). Looking at the seasonal variation, it is clear that winter and autumn have reduced data availability in general. In these seasons relatively fewer offshore stations are visited. Both are due to the harsh weather conditions in these seasons.

Moreover, external influences caused the sampling intensity to fluctuate over the years. These influences mainly being the downscaling of funding and later the upscaling of funding, creating data gaps of varying extent (Fig. 4). Two important data gaps are seen as a result of downscaled funding: a first in the nutrient data series, between 2007 and 2009; and a second for SPM measurements in 2008. Over the complete data series several shorter periods of fluctuating sampling intensities are seen, especially in 2004 and 2005 when higher sampling intensities were recorded for nearshore stations 130, 120 and 330, due to recurrent measurements at the same station.

## Usage Notes

As the BPNS holds numerous habitats that hold protection from several statutes (e.g. Wetlands or Ramsar areas, Natura 2000 areas, Flemish nature reserves, areas of the decree of the Dunes, protected landscapes and the Flemish Ecological Network, Marine Spatial Plan, bird and habitats Directives) this scheme of sampling campaigns is considered as a significant tool in order to assess impacts on the above marine protected areas. Furthermore, this dataset provides relevant records that will enable interesting insights on the influences of human activities on the health of the coastal zone, as anthropogenic pressures are mainly visible in nutrient and pigment concentrations. Especially regarding the proximity of the Scheldt estuary and the important ship traffic that it involves due to the presence of the industrial harbours of Zeebrugge and Antwerp. Additional measurements in the future will give opportunities to investigate the extent of estuarine influence on offshore areas, which responds directly to the demands of both Water Framework Directive^9^ and Marine Strategy Framework Directive^9,10^. Furthermore, the occurrence of harmful algal blooms (HABs) with its associated negative impacts on water quality is becoming increasingly important as these events affect coastal tourism, recreation and aquaculture^28–30^. This dataset is important to assess or explain historic events, as well as to detect potential ongoing incidents. Finally, this dataseries provides invaluable supporting measurements for marine biodiversity and ecosystem research.

The provided quality control flagged potentially erroneous measurements, resulting in a reliable dataset. Despite the fact that the majority of the measurements received good quality labels, an important constraint is due to the changing quantifying methods, these methods measuring slightly different sets of parameters over time and should be carefully interpreted. Especially Violaxanthine, Phaeophytine, Anthoxanthine and Echinone were analysed irregularly and each parameter holds fewer than 500 measurements over the complete data series. Another important constraint is due to inadequate quantifying methods for nutrients in marine samples, during the period 2009–2013, resulting in imprecise measurements with large uncertainty ranges. The quality control accounted for these imprecise measurements and inaccurate values are flagged with a ‘probably bad data’ flag. For most of that time period, replicate samples were measured in parallel, using another more adequate quantifying method. As such, excluding imprecise nutrient data from the series, is not considered to have an impact. Another constraint is seen in the quality control flags, adopted from the controlled vocabularies developed by the BODC. After executing the quality control, it is not always possible to associate a specific flag, to a specific step in the conducted quality control. The difference between temporal outliers and trip outliers (both labelled probably good data); and the difference between data supplier issues and regional maximum/minimum outliers (both labelled probably bad data) cannot be seen. This constraint is not considered to have a major impact for potential users.

Despite recognized data gaps for some descriptors, the data series provides regular records over a period of fifteen years. The overall data series remains valuable for different univariate investigations. Due to the observed data gaps multivariate investigations over multiple years may be more problematic. The most optimal temporal series in terms of consistency comprises the period from 2014 to 2017. Spatial completion through seasonally averaged values would enable univariate as well as multivariate investigations over extended ecological gradients for these years. Since 2014 these data gaps are less frequent to absent, for the intensity of the sampling campaigns is reinforced since.

When using data from the LifeWatch Observatory^18–20^, please use the following acknowledgements: ‘Nutrient, pigment and turbidity data were provided as part of the Flemish contribution to the LifeWatch ESFRI by Flanders Marine Institute (VLIZ).’

### ISA-Tab metadata file

Download metadata file

## Data Availability

Figures 1, 3 and 4 are generated with R (https://www.r-project.org/). The written codes to generate these figures are publically accessible via a fixed repository^25^. This repository contains both scripts and data files to allow users to fully comprehend the provided figures. The bathymetry raster used in Figs 1 and 3 are based on the EMODnet bathymetry maps^26^.

## Online-only Tables

**Online only Table 1:** Summary of parameter groups, parameters, units and count of measurements

Parameter group	Parameter	Unit	Number of records
Nutrients	Ammonium NH4	µmol N NH4/L	1507
	Nitrate Nitrite	µmol N NO3-NO2/L	1213
	Nitrate NO3	µmol N NO3/L	1275
	Nitrite NO2	µmol N NO2/L	1786
	Phosphate PO4	µmol P PO4/L	1794
	Silicate SiO4	µmol Si SiO4/L	1776
Pigments	19But Fucoxanthin	µg/l	1022
	19Hex Fucoxanthin	µg/l	880
	Alloxanthin	µg/l	1591
	Antheraxanthin	µg/l	717
	Anthoxanthin	µg/l	224
	Beta Carotene	µg/l	1081
	Chlorophyll a	µg/l	1594
	Chlorophyll b	µg/l	1593
	Chlorophyll c2	µg/l	1058
	Chlorophyll c2-c1	µg/l	667
	Chlorophyll c3	µg/l	1588
	Chlorophyllide a	µg/l	876
	Diadinoxanthin	µg/l	1593
	Diatoxanthin	µg/l	1592
	Echinenone	µg/l	340
	Fucoxanthin	µg/l	1593
	Lutein	µg/l	997
	Neoxanthin	µg/l	1270
	Peridinin	µg/l	1564
	Pheophorbide a	µg/l	892
	Pheophytin a	µg/l	967
	Pheophytin b	µg/l	141
	Prasinoxanthin	µg/l	784
	Violaxanthin	µg/l	339
	Zeaxanthin	µg/l	1451
SPM	SPM	mg/l	1540
Turbidity	Secchi depth	cm	1627
